# Estimated glomerular filtration rate may be an independent predictor for clinical outcomes regardless of acute kidney injury complication in the emergency department

**DOI:** 10.1371/journal.pone.0258665

**Published:** 2021-10-14

**Authors:** Ryohei Horie, Yuri Endo, Kent Doi

**Affiliations:** Department of Acute Medicine, The University of Tokyo Hospital, Bunkyo, Tokyo, Japan; University of Sao Paulo Medical School, BRAZIL

## Abstract

**Study objective:**

Acute kidney injury (AKI), chronic kidney disease (CKD), and decreased estimated glomerular filtration rate (eGFR) are all associated with poor clinical outcomes among emergency department (ED) patients. This study aimed to evaluate the effect of different types of renal dysfunction and the degree of eGFR reduction on the clinical outcomes in a real-world ED setting.

**Methods:**

Adult patients with an eGFR lower than 60 mL/min/1.73m^2^ in our ED, from October 1, 2016, to December 31, 2016, were enrolled in this retrospective observational study. Besides AKI and CKD, patients with unknown baseline renal function before an ED visit were categorized in the undetermined renal dysfunction (URD) category.

**Results:**

Among 1495 patients who had eGFR evaluation at ED, this study finally enrolled 441 patients; 22 patients (5.0%) had AKI only, 32 (7.3%) had AKI on CKD, 196 (44.4%) had CKD only, 27 (6.1%) had subclinical kidney injury (those who met neither criteria for AKI nor CKD), and 164 (37.2%) had URD. There was a significant association between eGFR and critical illness defined as the composite outcome of death or intensive care unit (ICU) need, hospitalization, ICU need, death, and renal replacement therapy need (odds ratio [95% confidence interval]: 1.72 [1.45–2.05], 1.36 [1.16–1.59], 1.66 [1.39–2.00], 1.73 [1.32–2.28], and 2.71 [1.73–4.24] for every 10 mL/min/1.73m^2^ of reduction, respectively). Multivariate logistic regression analysis showed eGFR was an independent predictor of critical illness composite outcome (death or ICU need), hospitalization, and ICU need even after adjustment with AKI or URD.

**Conclusions:**

Estimated GFR may be a sufficient predictor of clinical outcomes of ED patients regardless of AKI complication. Considerable ED patients were determined as URD, which might have a significant impact on the ED statistics regarding renal dysfunction.

## Introduction

Acute kidney injury (AKI) is a common condition among patients visiting the emergency departments (ED). A meta-analysis published in 2013 showed that the pooled incidence rates of AKI in adults were 21.6% worldwide [1]. Multiple studies have revealed that AKI is common and associated with poor clinical outcomes in EDs [2–4], emergency hospitalization [5], hospital wards [6], and intensive care units (ICUs) [7–9]. AKI development is also associated with a higher mortality rate in various clinical contexts, including sepsis [10–12], cardiogenic shock [13], chest pain [14], gunshot wound [15], emergent laparotomy [16], and out-of-hospital cardiac arrest [17].

Conversely, another clinical presentation of kidney disease, chronic kidney disease (CKD), is also known to be frequently observed in EDs. The global prevalence of CKD was reported to be as high as 11%–13% [18]. CKD patients commonly present to EDs, and those who have more advanced CKD are more likely to visit an ED for acute care [19]. Even for those without renal disease-specific symptoms, the ED could be a good setting to capture undiagnosed CKDs [20]. Although the information on the clinical impact of overlapping AKI and CKD is still limited, AKI superimposed on CKD has been suggested to be associated with worse outcomes than the isolated AKI [21–23].

AKI is diagnosed mainly when the serum creatinine value increases from the baseline [24]. CKD is a renal abnormality lasting more than three months, characterized by reduced estimated glomerular filtration rate (eGFR) or proteinuria [25]. Based on these diagnostic criteria, theoretically, AKI and CKD differentiation requires multiple measurements of renal function in one patient. However, obtaining the baseline value of the renal function in EDs is often difficult, and therefore, discrimination of AKI from CKD is difficult using a single serum creatinine value. In one study, 11% of emergently admitted patients with reduced eGFR did not have baseline data for renal function [26]. More recently, the severity of eGFR reduction in one-time ED measurement was reportedly associated with the higher 30-day mortality rate [27]. Although the diagnostic value of a single measurement of eGFR in EDs is yet to be validated with strong levels of evidence, multiple studies assessed an eGFR measured in the ED in various clinical settings, which was shown to be associated with the clinical outcomes such as mortality [27–30]. However, whether the magnitude of eGFR impairment regardless of AKI or CKD complications could have a higher impact on the outcome than accurate evaluation of renal dysfunction, e.g., AKI versus CKD, has not been addressed well.

This retrospective study aimed to describe the actual epidemiology and characteristics of renal dysfunction of ED patients, including AKI, CKD, and undetermined type due to lack of baseline information and to evaluate the impact of eGFR reduction and types of renal dysfunction on the clinical outcomes in the real-world ED setting.

## Methods

### Study design and setting

This single-center, retrospective, observational study took place in the ED of the University of Tokyo Hospital. It is a tertiary medical center located in an urban area of Tokyo, Japan, with approximately 12,000 annual emergency visits. The institutional review board at the University of Tokyo Hospital approved this retrospective study and waived informed consent [#3820-(2)].

### Study protocol

Adult patients presented to the ED between October 1, 2016, to December 31, 2016, were retrospectively reviewed for study enrollment. The inclusion criteria were as follows: 1) patients older than 18 years and 2) for whom a blood test was performed with eGFR lower than 60 mL/min/1.73m^2^ at the time of ED visit. When a patient had multiple blood tests in 1 day, the first test during the ED visit was set as the reference. To describe the adult ED population comprehensively, the key characteristics of adult patients who did not meet criterion 2) were still recorded and summarized unless meeting the following exclusion criteria (screening analysis). The exclusion criteria included the following: 1) patients who presented to ED with out-of-hospital cardiac arrest, 2) patients who had had a known end-stage renal disease and had been on a maintenance renal replacement therapy (RRT) before the ED visit, and 3) patients who eventually transferred to another hospital on the day of the ED visit. When a single patient had multiple ED visits during the study period, we analyzed the data from the earliest visit to avoid patient duplication. The serum creatinine and eGFR values from the last available blood test obtained between 7 days and 1 year prior to the ED encounter were considered the baseline renal function. Patients with missing variables on the day of ED visit were excluded, although those who had no baseline renal function were still included in the study.

### Measurements

Clinical data including demographics, blood test results including serum creatinine and eGFR at the time of ED presentation and at the time of the last measurement between 7 days and 1 year before the ED encounter (baseline), initial vital signs (systolic blood pressure, body temperature, and level of consciousness) in ED, chief complaints about ED visit, and important medical conditions such as sepsis, types of infections, and abnormal body temperature were extracted from the electronic medical record and were reviewed by two emergency physicians. Body mass index was also recorded when available. Altered mental status (AMS) was defined as a Glasgow Coma Scale lower than 15. For both of ED and baseline values, serum creatinine levels were measured at the central laboratory of our hospital by enzymatic method with LABOSPECT 008α (Hitachi High-Tech^®^, reference ranges: 0.65–1.07 mg/dL for male and 0.46–0.79 mg/dL for female). The Modified Diet Renal Disease equation for Japanese patients [31] was used for eGFR calculation. Referring to the Sequential Organ Failure Assessment (SOFA) score [32], known predictors of poor clinical outcomes (platelet counts and total bilirubin levels on the day of ED visit) were also collected. For all patients, clinical outcomes, including hospitalization, hospital death, need of ICU treatment, and RRT, were recorded.

The patient’s renal dysfunction was categorized into the following: “AKI only,” “CKD only,” “AKI on CKD,” “subclinical kidney injury,” or “undetermined renal dysfunction (URD).” Patients who had baseline data for their renal function were assessed for the presence of AKI or CKD. AKI and CKD were diagnosed based on the criteria shown in the KDIGO guidelines [24, 25]. AKI was diagnosed when a patient’s ED creatinine had increased from the baseline by greater than 0.3 mg/dL or 50%. CKD was diagnosed when a patient had a baseline eGFR lower than 60 mL/min/1.73m^2^. “AKI on CKD” was diagnosed when a patient simultaneously met the criteria for AKI and CKD. When a patient did not have 0.3 mg/dL or 50% increase of serum creatinine from the baseline, and the baseline eGFR was greater than 60 mL/min/1.73m^2^, that patient did not meet the criteria for any of AKI and CKD. Such patients were categorized into the “subclinical kidney injury” because the eGFR at ED visit was below 60 mL/min/1.73m^2^. Those who had no data of baseline renal function were coded as “URD.”

### Outcomes

The primary outcome was critical illness, defined as the composite endpoint of hospital death or ICU need. Some critically ill patients were assumed to decline from ICU transfer due to advanced ages or other underlying medical conditions such as advanced cancer, these patients still would have a high mortality rate. The purpose of setting the above primary endpoint is to comprehensively capture these severely ill patients in the analysis. As the secondary outcomes, hospitalization, the need for ICU, in-hospital death, and the need for RRT were evaluated.

### Data analysis

All statistical analyses were performed using JMP^®^ Pro 15.2.1 (2019 SAS institute, all right reserved). Data were shown in number with percentage for categorical variables and median with interquartile range (IQR) for continuous variables. Continuous variables in multiple categories were compared using ANOVA or Wilcoxon test depending on the skewness of the data. Categorical variables were compared using the chi-square test or Fisher’s exact test as appropriate.

Univariate and multivariate logistic regression analyses were performed to evaluate the effect of the independent variables on the outcomes and were reported with an odds ratio with 95% confidence intervals and p-values. Multivariate analysis was completed only for those outcomes with a sufficient number of positive cases. Predefined independent variables were used for multivariate logistic regression; these were the presence of AKI, presence of URD, eGFR in ED, low platelet count (<150 × 10^3^/μL), elevated total bilirubin (≥1.2 mg/dL), presence of AMS, and age of > 75 years old. Since a previous study has reported that the degree of eGFR reduction and mortality had a quantitative relationship [27], eGFR was treated as a continuous variable in a logistic regression model. For all statistical analyses, a p-value of less than 0.05 was considered statistically significant.

As sensitivity analyses, the above univariate and multivariate logistic regression analyses were performed using different formulas for eGFR calculation (original MDRD [33] and CKD-EPI [34]). For this purpose, the eGFR of each patient was re-calculated from the serum creatinine value of each time point (baseline and ED), and the inclusion and the exclusion criteria for the analyses were re-applied. Renal dysfunction of each patient was re-categorized based on the re-calculated eGFR values by two different formulas.

For those patients whose BMI values were available, univariate logistic regression analyses for the primary outcome in different BMI subgroups (BMI < 18.5, 18.5 ≦ BMI < 25, and BMI ≧ 25) were performed. Due to small numbers of positive patients in each BMI category, multivariate logistic regression analyses were not performed for this purpose.

To assess the accuracy of continuous independent variables for the prediction of the binary outcomes, receiver operating characteristics (ROC) curves were generated, and the areas under curves were shown with 95% confidence intervals. For those variables with an area under the curve of greater than 0.7, the best cutoffs were determined using the Youden index.

## Results

**Fig 1:**
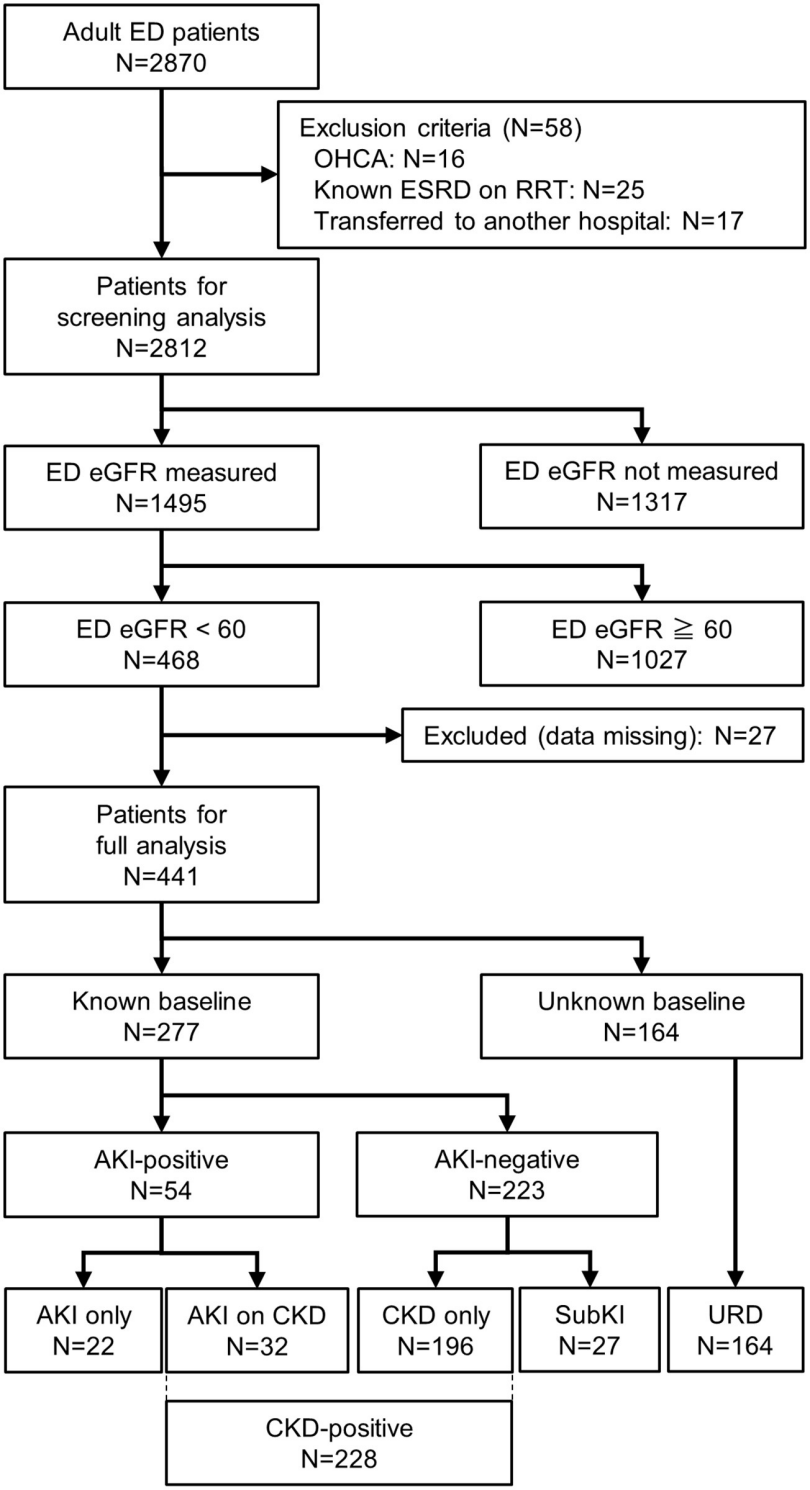
Patient enrollment and categorization. ED, emergency department; eGFR, estimated glomerular filtration rate; OHCA, out-of-hospital cardiac arrest; ESRD, end-stage renal disease; RRT, renal replacement therapy; AKI, acute kidney injury; CKD, chronic kidney disease; SubKI, subclinical kidney injury; URD, undetermined renal disfunction.

During the study period, a total of 2,870 adult patients presented to the ED. After excluding 58 patients (16 patients presenting with out-of-hospital cardiac arrest, 25 patients with end stage renal disease on RRT, 17 patients who were directly transferred to another hospital for immediate treatment), 2,812 patients were qualified for the screening analysis. There were 1,317 patients whose serum creatinine was not measured during the ED visit, and 1,495 patients underwent at least one ED measurement of eGFR. Among these patients, 1,027 had an eGFR ≧ 60 mL/min/1.73m^2^ and 468 had an eGFR < 60 mL/min/1.73m^2^. Additionally, 27 patients were excluded due to missing data and 441 patients were qualified for the full analysis (Fig 1).

The serum creatinine and eGFR values from the last available blood test obtained between 7 days and 1 year prior to the ED encounter were considered the baseline. AKI and CKD were diagnosed based on KDIGO guidelines [24, 25]. When a patient whose baseline renal function was available did not meet the criteria for AKI or CKD, it was categorized as subclinical kidney injury. Patients without the baseline information were categorized into undetermined renal dysfunction.

**Fig 2:**
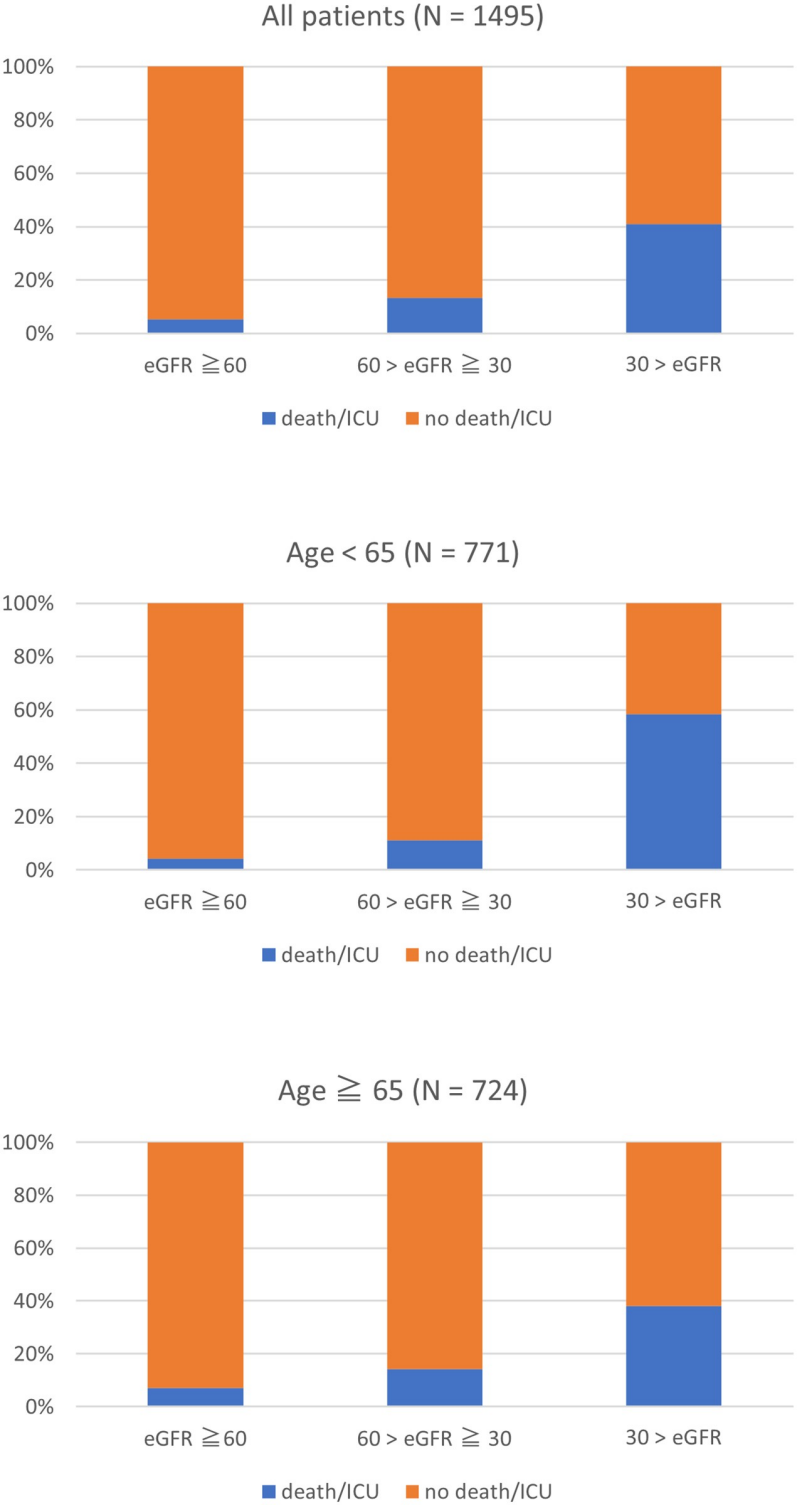
Critical illness and eGFR ranges. eGFR, estimated glomerular filtration rate; ICU, intensive care unit. Critical illness was defined as the composite endpoint of death or ICU requirement.

Table 1 summarizes the characteristics of all adult patients screened for the study. Patients with an ED eGFR < 60 mL/min/1.73m^2^ tended to be older as compared to those with higher eGFR or those who did not undergo eGFR measurement. The vast majority of the patients without an eGFR measurement did not require hospitalization (only 1.4% of them were hospitalized). In contrast, the patients with ED eGFR < 60 mL/min/1.73m^2^ were relatively sicker, with 18.6% of critical illness (death or ICU need) and 59.8% of hospitalization. The rate of critical illness of the patients by different eGFR range is shown in Fig 2. There was a tendency of the stepwise increase in the rate of critically ill patients associated with ED eGFR decrease in overall, younger, and older groups. The full data of the screened patients are available in S1 Table.

**Table 1:** Screening analysis: All adult ED patients.

Variable	Overall (N = 2812)	ED eGFR		
		Not measured (n = 1317)	≧60 mL/min/1.73m^2^ (n = 1027)	< 60 mL/min/1.73m^2^ (n = 468)
Age, y, median (IQR)	54 (36, 74)	44 (31, 67)	52 (36, 70)	77 (66, 84)
Sex, male (%)	1366 (48.6)	600 (45.6)	505 (49.2)	261 (55.8)
**Renal function, Median (IQR)**				
ED creatinine, mg/dL	0.76 (0.61, 0.97)	not measured	0.66 (0.56, 0.79)	1.13 (0.95, 1.44)
ED eGFR, mL/min/1.73m^2^	73.6 (54.4, 91.7)	not measured	84.4 (72.7, 98.1)	45.7 (34.2, 53.3)
**Outcome, no. (%)**				
Death or ICU	144 (5.1)	3 (0.2)	54 (5.3)	87 (18.6)
Hospitalized	736 (26.2)	19 (1.4)	437 (42.6)	280 (59.8)
ICU needed	125 (4.4)	2 (0.2)	49 (4.8)	74 (15.8)
Hospital death	34 (1.2)	1 (0.1)	8 (0.8)	25 (5.3)

*IQR*, interquartile range; *ED*, emergency department; *ICU*, intensive care unit; *eGFR*, estimated glomerular filtration rate

Among the fully analyzed 441 patients, 243 were male (55.1%), and the median age was 77 years (IQR: 66.5 to 84). Regarding clinical outcomes, critical illness (defined as death or ICU need) was observed in 86 patients (19.5%), 273 (61.9%) were hospitalized, 73 (16.6%) required ICU care during their hospital stay, and 25 died (5.7%) in hospital. The number of patients needing intermittent or continuous RRT was 12 (2.7%) (Table 2). The baseline serum creatinine values were missing in 164 patients (37.2%). BMI values were missing in 112 patients (25.4%). The patients showed various chief complaints, and systemic complaints, such as fever or generalized fatigue, were the most common. A small proportion of patients presented with direct kidney-related problems, such as abnormal electrolytes. Sepsis was suspected in 6.8%. In terms of common source of infections, pneumonia and urinary tract infection were present in 9.3% and 6.6% respectively. Influenza was not commonly seen during the study period. About one fourth of the patients had body temperature of ≧38°C or < 36°C. Full data on the study participants are available in S2 Table.

**Table 2:** Full analysis: Summary of the patient characteristics, clinical variables, and outcomes.

Variable	Total (N = 441)	Missing data, No. (%)
Age, y, median (IQR)	77 (66.5, 84)	0 (0)
Sex, male, No. (%)	243 (55.1)	0 (0)
**Reason for ED visit, no. (%)**		
Systemic	103 (23.4)	0 (0)
Abdominal	86 (19.5)	0 (0)
Neurological	86 (19.5)	0 (0)
Cardiovascular	49 (11.1)	0 (0)
Respiratory	41 (9.3)	0 (0)
Trauma	41 (9.3)	0 (0)
Other	35 (7.9%)	0 (0)
**Clinical variable, median (IQR)**		
Baseline creatinine, mg/dL	1.04 (0.87, 1.33)	164 (37.2)
Baseline eGFR, mL/min/1.73m^2^	47.8 (38.6, 55.9)	164 (37.2)
ED creatinine, mg/dL	1.12 (0.94, 1.42)	0 (0)
ED eGFR, mL/min/1.73m^2^	45.7 (34.6, 53.3)	0 (0)
Systolic blood pressure, mmHg	131 (112, 152)	0 (0)
Body temperature, °C	36.7 (36.2, 37.1)	0 (0)
Glasgow Coma Scale	15 (14, 15)	0 (0)
Platelet count, × 10^3^/μL	205 (154, 253)	0 (0)
Total bilirubin, mg/dL	0.7 (0.5, 1.0)	0 (0)
Body mass index, kg/m^2^	22.1 (19.3, 24.7)	112 (25.4)
**Medical conditions, no. (%)**		
Sepsis	30 (6.8)	0 (0)
Pneumonia	41 (9.3)	0 (0)
Urinary tract infection	29 (6.6)	0 (0)
Influenza	4 (0.9)	0 (0)
Other infection	61 (13.8)	0 (0)
Environmental hyperthermia	1 (0.2)	0 (0)
Environmental hypothermia	1 (0.2)	0 (0)
Body temperature ≧ 38°C	55 (12.5)	0 (0)
Body temperature < 36°C	61 (13.8)	0 (0)
**Clinical outcome, no. (%)**		
Death or ICU	86 (19.5)	0 (0)
Hospitalization	273 (61.9)	0 (0)
ICU need	73 (16.6)	0 (0)
Hospital death	25 (5.7)	0 (0)
RRT need	12 (2.7)	0 (0)

*IQR*, interquartile range; *ED*, emergency department; *ICU*, intensive care unit; *RRT*, renal replacement therapy; *eGFR*, estimated glomerular filtration rate

**Fig 3:**
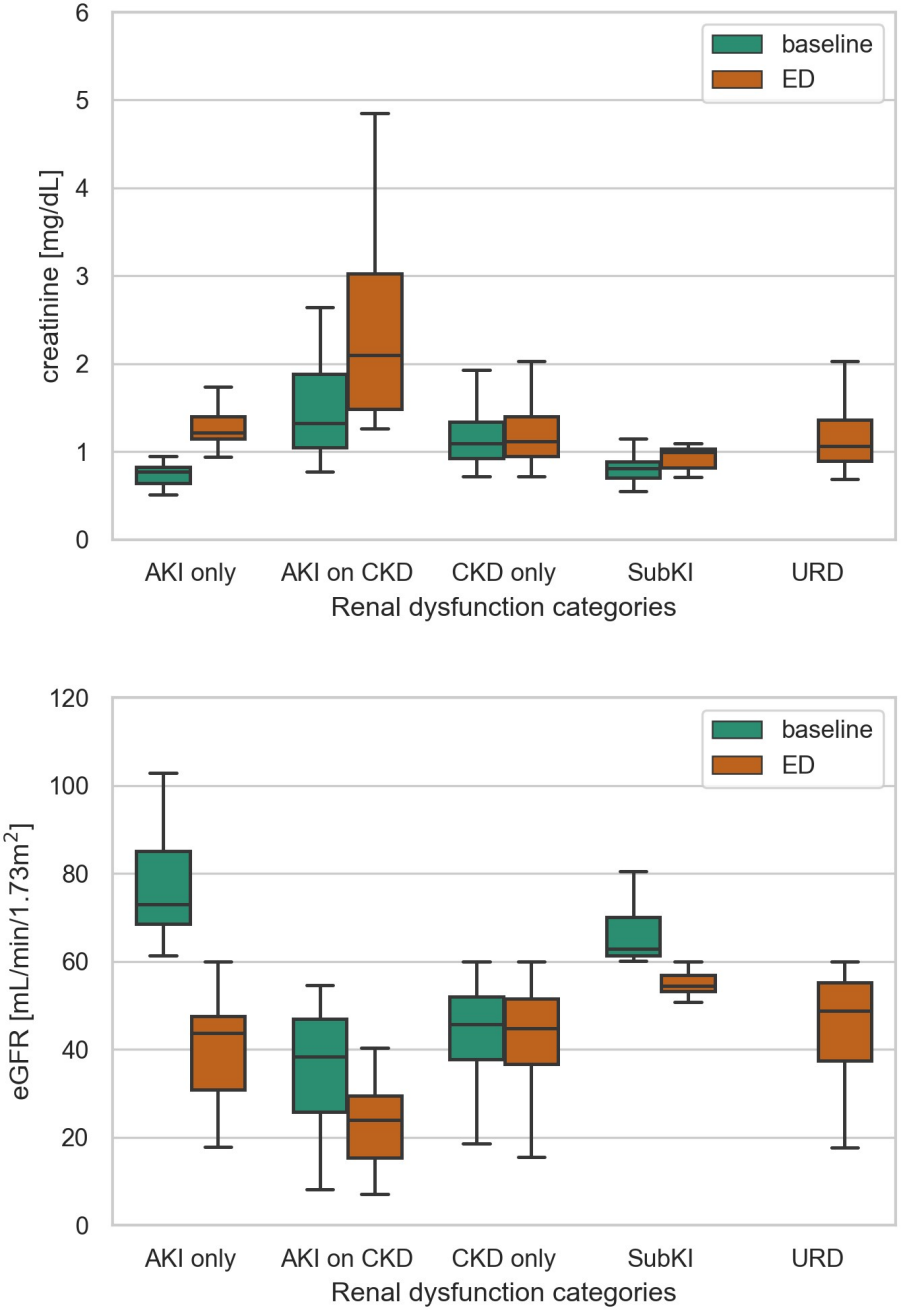
Baseline and ED measurement of creatinine and eGFR. AKI, acute kidney injury; CKD, chronic kidney disease; SubKI, subclinical kidney injury; URD, undetermined renal dysfunction; eGFR, estimated glomerular filtration rate. By definition, there is no baseline measurement of creatinine or eGFR for URD.

Table 3 shows the patient distribution in each category of renal dysfunction. The patients with “CKD only” were the most common, followed by those with URD. A statistically significant difference was observed in age, baseline and ED renal functions, body temperature, and AMS among these categories, although the difference in body temperature seemed clinically meaningless. Patients in the AKI on CKD group were the oldest, while those in the AKI only group were the youngest. The difference of serum creatine and eGFR between baseline and ED visit was the largest in the AKI on CKD group and AKI only group, respectively. In contrast, there was virtually no significant difference in the CKD only group (Fig 3). Table 3 also summarizes the clinical outcomes among renal dysfunction categories. AKI was significantly associated with poor outcomes regarding the composite outcome of death or ICU requirement, hospitalization, ICU requirement, hospital death, and RRT. Notably, ICU treatment was commonly required in the URD group (23.2%), whereas the hospital death rate in this group was only 3.0%, which was lower than the overall death rate. Regarding the composite outcome of death and ICU requirement, the URD group showed the second-highest positive rate. Based on these findings, five groups were recategorized into three groups for logistic regression analyses: AKI-positive (AKI only and AKI on CKD), AKI-negative (CKD only and subclinical kidney injury), and URD. The univariate analysis also proved these associations, as shown in Table 4.

**Table 3:** Summary of demographics, clinical variables, and outcomes by different types of renal dysfunction.

Variable	Overall (N = 441)	Types of renal dysfunction					p-value
		AKI only (n = 22)	AKI on CKD (n = 32)	CKD only (n = 196)	SubKI (n = 27)	URD (n = 164)	
Age, y, median (IQR)	77 (66.5, 84)	70.5 (60, 75.8)	79.5 (70, 85.5)	78 (68.3, 84)	72 (63, 81)	77 (64, 84)	**0.0106**
Sex, male (%)	243 (55.1)	15 (68.2)	19 (59.4)	103 (52.6)	15 (55.6)	91 (55.5)	0.6835
**Clinical variable, median (IQR)**							
Baseline creatinine, mg/dL	1.04 (0.87, 1.33)	0.77 (0.63, 0.83)	1.32 (1.03, 1.92)	1.09 (0.92, 1.34)	0.81 (0.69, 0.90)	NA^a^	**< 0.0001**
Baseline eGFR, mL/min/1.73m^2^	47.8 (38.6, 55.9)	73.0 (68.0, 85.8)	38.3 (25.3, 47.4)	45.7 (37.5, 52.0)	62.9 (61.2, 71.1)	NA^a^	**< 0.0001**
ED creatinine, mg/dL	1.12 (0.94, 1.42)	1.22 (1.15, 1.49)	2.10 (1.48, 3.34)	1.12 (0.95, 1.40)	0.99 (0.81, 1.03)	1.07 (0.89, 1.36)	**< 0.0001**
ED eGFR, mL/min/1.73m^2^	45.7 (34.6, 53.3)	43.7 (29.1, 47.5)	23.9 (14.8, 30.0)	44.7 (36.4, 51.6)	54.5 (53.1, 57.3)	48.8 (37.1, 55.4)	**< 0.0001**
Systolic blood pressure, mmHg	131 (112, 152)	110 (103, 133)	128 (106, 146)	131 (119, 152)	121 (100, 150)	133 (112, 157)	0.1711
Body temperature, °C	36.7 (36.2, 37.1)	36.8 (36.2, 37.6)	36.7 (36.1, 37.3)	36.7 (36.3, 37.5)	36.4 (36.2, 37.4)	36.5 (36.0, 37.0)	**0.0028**
Glasgow Coma Scale	15 (14, 15)	15 (14.8, 15)	15 (15, 15)	15 (15, 15)	15 (15, 15)	15 (14, 15)	**0.0092**
Platelet count, × 10^3^/μL	205 (154, 253)	193 (132, 251)	189 (120, 239)	198 (147, 260)	216 (163, 287)	213 (168, 249)	0.248
Total bilirubin, mg/dL	0.7 (0.5, 1.0)	1.0 (0.4, 2.3)	0.7 (0.4, 1.1)	0.7 (0.5, 1.0)	0.7 (0.5, 1.0)	0.6 (0.5, 0.9)	0.1671
AMS^b^, no (%)	115 (26.1)	5 (22.7)	7 (21.9)	41 (20.9)	4 (14.8)	58 (35.4)	**0.0189**
**Outcome, no. (%)**							
Death or ICU	86 (19.5)	5 (22.7)	11 (34.4)	29 (14.8)	2 (7.4)	39 (23.8)	**0.0185**
Hospitalization	273 (61.9)	19 (86.4)	25 (78.1)	114 (58.2)	19 (70.4)	96 (58.5)	**0.0179**
ICU need	73 (16.6)	4 (18.2)	9 (28.1)	20 (10.2)	2 (7.4)	38 (23.2)	**0.0035**
Hospital death	25 (5.7)	4 (18.2)	5 (15.6)	11 (5.6)	0 (0)	5 (3.0)	**0.0029**
RRT need	12 (2.7)	2 (9.1)	3 (9.4)	3 (1.5)	0 (0)	4 (2.4)	**0.0317**

Significant p-values are shown in bold.

*AKI*, acute kidney injury; *CKD*, chronic kidney disease; *SubKI*, subclinical kidney injury; *URD*, undetermined renal dysfunction; *IQR*, interquartile range; *ED*, emergency department; *ICU*, intensive care unit; *RRT*, renal replacement therapy; *eGFR*, estimated glomerular filtration rate; *AMS*, altered mental status.

^a^ By definition, there is no baseline measurement of creatinine or eGFR for URD.

^b^ AMS is defined as Glasgow Coma Scale of less than 15.

**Table 4:** Univariate logistic regression model for factors associated with clinical outcomes.

Outcome, positive cases (%)	Patient factor	Reference	p-value	OR (95% CI)
**Death or ICU 86 (19.5)**	AKI-positive	AKI-negative	**0.007**	2.61 (1.30–5.23)
	URD	AKI-negative	**0.0135**	1.93 (1.15–3.26)
	eGFR	N/A^a^	**<0.0001**	1.72 (1.45–2.05)
	SBP ≥180mmHg	SBP 90–180mmHg	**0.0396**	2.40 (1.04–5.52)
	SBP < 90mmHg	SBP 90–180mmHg	**<0.0001**	6.04 (2.86–12.76)
	BT ≥38°C	BT 36°C–38°C	**0.001**	2.90 (1.54–5.46)
	BT < 36°C	BT 36°C–38°C	**0.0201**	2.12 (1.13–4.01)
	Platelet count < 150 × 10^3^/μL	Platelet count ≥150 × 10^3^/μL	**<0.0001**	2.95 (1.78–4.89)
	Total bilirubin ≥1.2 mg/dL	Total bilirubin < 1.2 mg/dL	0.0548	1.77 (0.99–3.15)
	GCS < 15	GCS = 15	**<0.0001**	4.19 (2.55–6.88)
	Age ≥75	Age < 75	0.3078	1.28 (0.79–2.07)
	Sex (male)	Sex (female)	0.9254	0.98 (0.61–1.57)
**Hospitalization 273 (61.9)**	AKI-positive	AKI-negative	**0.0037**	2.98 (1.42–6.22)
	URD	AKI-negative	0.8271	0.96 (0.63–1.44)
	eGFR	N/A^a^	**<0.0001**	1.36 (1.16–1.59)
	SBP ≥180mmHg	SBP 90–180mmHg	0.3571	1.47 (0.65–3.30)
	SBP < 90mmHg	SBP 90–180mmHg	0.1056	1.98 (0.87–4.52)
	BT ≥38°C	BT 36°C–38°C	**0.0007**	3.63 (1.72–7.67)
	BT < 36°C	BT 36°C–38°C	0.7494	1.10 (0.63–1.92)
	Platelet count < 150 × 10^3^/μL	Platelet count ≥150 × 10^3^/μL	**0.0123**	1.86 (1.14–3.02)
	Total bilirubin ≥1.2 mg/dL	Total bilirubin < 1.2 mg/dL	**<0.0001**	3.66 (1.91–7.04)
	GCS < 15	GCS = 15	**0.0165**	1.76 (1.11–2.78)
	Age ≥75	Age < 75	0.7925	1.05 (0.72–1.55)
	Sex (male)	Sex (female)	0.0911	1.39 (0.95–2.05)
**ICU need 73 (16.6)**	AKI-positive	AKI-negative	**0.0063**	2.90 (1.35–6.22)
	URD	AKI-negative	**0.0005**	2.76 (1.56–4.87)
	eGFR	N/A^a^	**<0.0001**	1.66 (1.39–2.00)
	SBP ≥180mmHg	SBP 90–180mmHg	**0.0112**	2.97 (1.28–6.89)
	SBP < 90mmHg	SBP 90–180mmHg	**<0.0001**	5.13 (2.40–10.97)
	BT ≥38°C	BT 36°C–38°C	0.0761	1.88 (0.94–3.76)
	BT < 36°C	BT 36°C–38°C	0.0851	1.81 (0.92–3.54)
	Platelet count < 150 × 10^3^/μL	Platelet count ≥150 × 10^3^/μL	**0.0003**	2.66 (1.56–4.54)
	Total bilirubin ≥1.2 mg/dL	Total bilirubin < 1.2 mg/dL	0.4713	1.27 (0.66–2.42)
	GCS < 15	GCS = 15	**<0.0001**	3.09 (1.83–5.21)
	Age ≥75	Age < 75	0.7096	1.10 (0.66–1.83)
	Sex (male)	Sex (female)	0.7524	0.92 (0.56–1.53)
**Hospital death 25 (5.7)**	AKI-positive	AKI-negative	**0.0048**	3.85 (1.51–9.85)
	URD	AKI-negative	0.3621	0.61 (0.21–1.78)
	eGFR	N/A^a^	**<0.0001**	1.73 (1.32–2.28)
	SBP ≥ 180mmHg	SBP 90–180mmHg	0.9887	<0.1 (0–99)
	SBP < 90mmHg	SBP 90–180mmHg	**0.0038**	4.38 (1.61–11.92)
	BT ≥38°C	BT 36°C–38°C	**0.0264**	3.19 (1.15–8.90)
	BT < 36°C	BT 36°C–38°C	**0.0144**	3.38 (1.27–8.97)
	Platelet count < 150 × 10^3^/μL	Platelet count ≥ 150 × 10^3^/μL	**0.0002**	4.74 (2.08–10.81)
	Total bilirubin ≥ 1.2 mg/dL	Total bilirubin < 1.2 mg/dL	**0.0004**	4.57 (1.98–10.54)
	GCS < 15	GCS = 15	**<0.0001**	6.90 (2.89–16.46)
	Age ≥75	Age < 75	**0.0164**	3.40 (1.25–9.22)
	Sex (male)	Sex (female)	0.6122	1.24 (0.54–2.82)
**RRT need 12 (2.7)**	AKI-positive	AKI-negative	**0.0071**	7.48 (1.73–32.37)
	URD	AKI-negative	0.4316	1.83 (0.40–8.31)
	eGFR	N/A^a^	**<0.0001**	2.71 (1.73–4.24)
	SBP ≥180mmHg	SBP 90–180mmHg	**0.0019**	7.44 (2.10–26.41)
	SBP < 90mmHg	SBP 90–180mmHg	0.9889	<0.1 (0–99)
	BT ≥38°C	BT 36°C–38°C	0.617	1.50 (0.31–7.23)
	BT < 36°C	BT 36°C–38°C	0.7133	1.34 (0.28–6.48)
	Platelet count < 150 × 10^3^/μL	Platelet count ≥150 × 10^3^/μL	0.1342	2.44 (0.76–7.87)
	Total bilirubin ≥1.2 mg/dL	Total bilirubin < 1.2 mg/dL	0.4586	0.46 (0.06–3.61)
	GCS < 15	GCS = 15	0.5636	1.43 (0.42–4.85)
	Age ≥75	Age < 75	0.6951	0.79 (0.25–2.50)
	Sex (male)	Sex (female)	0.4083	1.65 (0.49–5.57)

Significant p-values are shown in bold.

*OR*, odds ratio; *CI*, confidence interval; *ICU*, intensive care unit; *RRT*, renal replacement therapy; *AKI*, acute kidney injury; *URD*, undetermined renal dysfunction; *eGFR*, estimated glomerular filtration rate; *SBP*, systolic blood pressure; *BT*, body temperature; *GCS*, Glasgow Coma Scale.

AKI-positive means those who were proven to have AKI, whereas AKI-negative means those who were proven NOT to have AKI based on the KDIGO guidelines. URD are the patients who could not be diagnosed with or ruled out AKI due to lack of an information on their baseline renal function.

^a^ For eGFR, OR for 10 mL/min/1.73m^2^ of eGFR decrease were shown.

Associations between patient factors and clinical outcomes were assessed using univariate and multivariate logistic regression. The eGFR in the ED consistently had a significant association with all the five outcomes in univariate analyses (Table 4) and three clinical outcomes in multivariate analyses (Table 5). The presence of AKI also showed a statistically significant association with all the five outcomes in univariate analyses (Table 4). However, multivariate analysis did not show a significant association of AKI presence with the clinical outcomes (Table 5). For URD, a significant association was observed with the composite outcome (death or ICU requirement) and ICU requirement in multivariate analysis (Table 5). However, no significant association was observed with hospital death (Table 4). Besides these renal factors, nonrenal factors including age, blood pressure, body temperature, presence of AMS, platelet count, and total bilirubin value also showed association with the outcomes as shown in Tables 4 and 5.

**Table 5:** Multivariate logistic regression model for factors associated with clinical outcomes.

Outcome, positive cases (%)	Patient factor	Reference	p-value	OR (95% CI)
**Death or ICU 86 (19.5)**	AKI-positive	AKI-negative	0.7076	1.17 (0.52–2.65)
	URD	AKI-negative	**0.0295**	1.93 (1.07–3.50)
	eGFR	N/A^a^	**<0.0001**	1.70 (1.38–2.09)
	Platelet count < 150 × 10^3^/μL	Platelet count ≥150 × 10^3^/μL	**0.0064**	2.34 (1.27–4.30)
	Total bilirubin ≥1.2 mg/dL	Total bilirubin < 1.2 mg/dL	0.3332	1.42 (0.70–2.88)
	GCS < 15	GCS = 15	**<0.0001**	4.06 (2.31–7.11)
	Age ≥75	Age < 75	0.3301	0.76 (0.43–1.32)
**Hospitalization 273 (61.9)**	AKI-positive	AKI-negative	0.1039	1.93 (0.87–4.28)
	URD	AKI-negative	0.959	0.99 (0.64–1.53)
	eGFR	N/A^a^	**0.0068**	1.27 (1.06–1.52)
	Platelet count < 150 × 10^3^/μL	Platelet count ≥150 × 10^3^/μL	0.3314	1.30 (0.77–2.20)
	Total bilirubin ≥1.2 mg/dL	Total bilirubin < 1.2 mg/dL	**0.0004**	3.37 (1.71–6.64)
	GCS < 15	GCS = 15	**0.0232**	1.77 (1.08–2.89)
	Age ≥75	Age < 75	0.494	0.87 (0.57–1.31)
**ICU need 73 (16.6)**	AKI-positive	AKI-negative	0.5062	1.34 (0.57–3.16)
	URD	AKI-negative	**0.0007**	2.96 (1.56–5.54)
	eGFR	N/A^a^	**<0.0001**	1.66 (1.34–2.05)
	Platelet count < 150 × 10^3^/μL	Platelet count ≥150 × 10^3^/μL	**0.0095**	2.33 (1.23–4.42)
	Total bilirubin ≥1.2 mg/dL	Total bilirubin < 1.2 mg/dL	0.9236	0.96 (0.44–2.08)
	GCS < 15	GCS = 15	**0.001**	2.67 (1.48–4.78)
	Age ≥75	Age < 75	0.2208	0.70 (0.39–1.24)

Significant p-values are shown in bold.

*OR*, odds ratio; *CI*, confidence interval; *ICU*, intensive care unit; *RRT*, renal replacement therapy; *AKI*, acute kidney injury; *URD*, undetermined renal dysfunction; *eGFR*, estimated glomerular filtration rate; *GCS*, Glasgow Coma Scale.

AKI-positive means those who were proven to have AKI, whereas AKI-negative means those who were proven NOT to have AKI based on the KDIGO guidelines. URD are the patients who could not be diagnosed with or ruled out AKI due to lack of an information on their baseline renal function.

^a^ For eGFR, OR for 10 mL/min/1.73m^2^ of eGFR decrease were shown.

**Fig 4:**
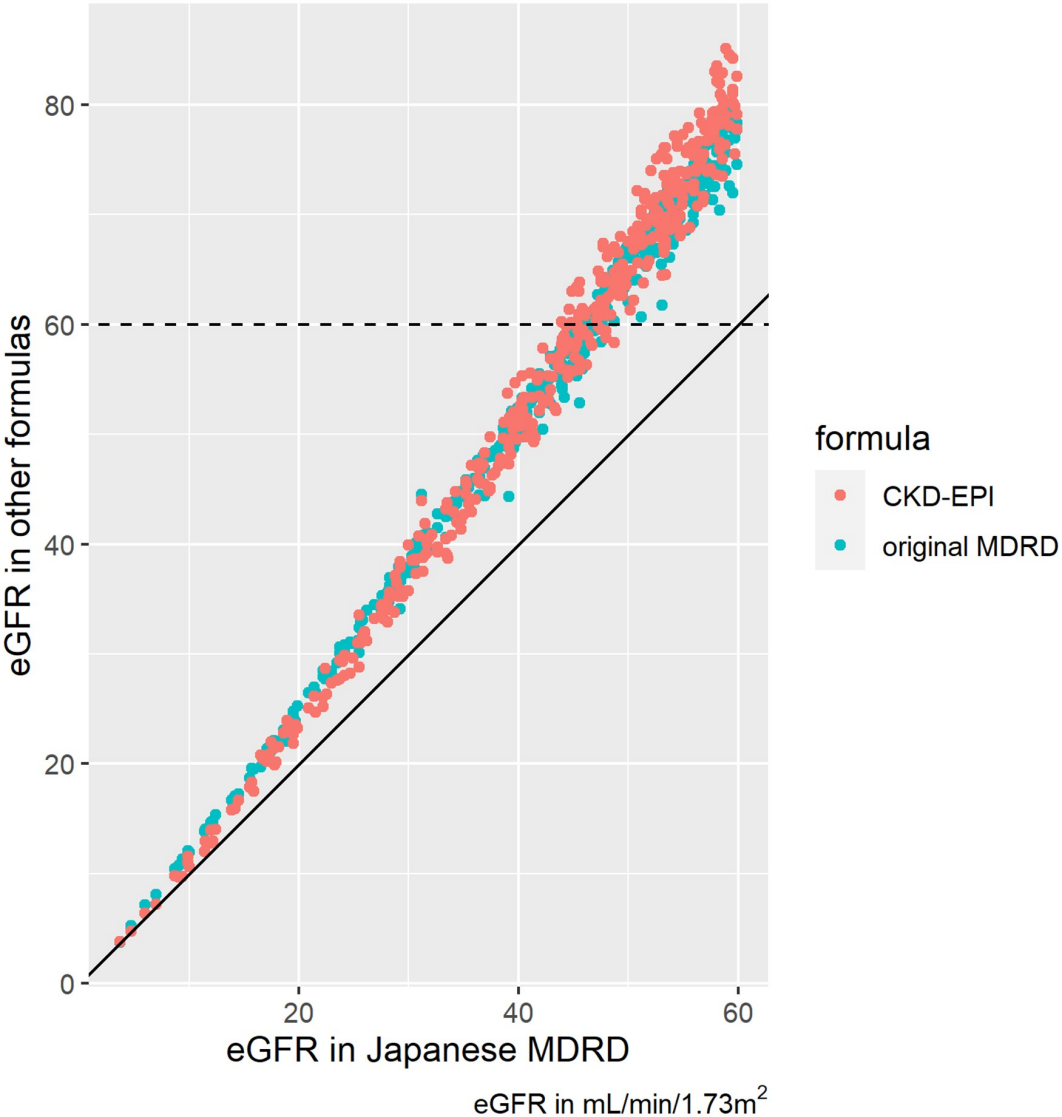
Comparison between eGFR by Japanese MDRD and other formulas. eGFR, estimated glomerular filtration rate. The solid line represents the level where Japanese MDRD equals to the other formula compared. The dashed line represents the eGFR value of 60 mL/min/1.73m^2^ by other formulas (original MDRD or CKD-EPI), which is the cutoff for the study enrollment. The patients above this dashed line would be excluded from the full analysis if eGFR were calculated with the other formulas (original MDRD or CKD-EPI).

**Fig 5:**
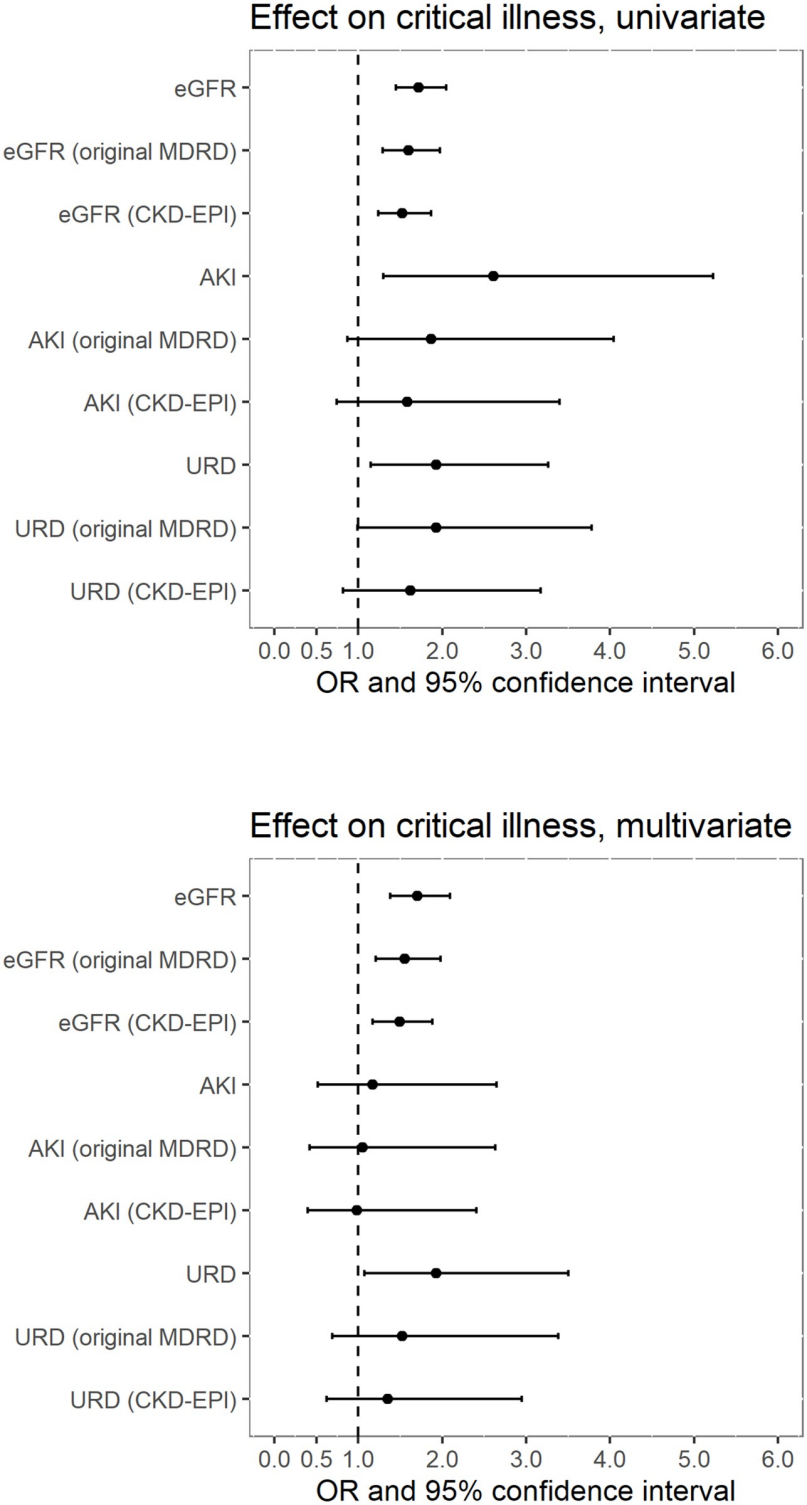
Effect sizes on critical illness in different eGFR formulas. OR, odds ratio; eGFR, estimated glomerular filtration rate; AKI, acute kidney injury; URD, undetermined renal dysfunction. For eGFR, OR for 10 mL/min/1.73m^2^ of eGFR decrease were shown.

As previously described, sensitivity analyses were performed using different eGFR formulas. Fig 4 compares the eGFR values by Japanese MDRD and other eGFR formulas. As compared to Japanese MDRD, original MDRD and CKD-EPI tended to give higher eGFR values. The difference between these formulas tended to be larger in those patients with higher eGFR values. Table 6 summarizes the characteristics of the patients enrolled in the full analysis using original MDRD and CKD-EPI. As expected from Fig 4, when original MDRD or CKD-EPI were used for eGFR calculation, less patients were enrolled in the full analysis. The median serum creatinine values were higher in original MDRD or CKD-EPI based enrollment. In terms of renal dysfunction categories, the numbers of patients in CKD only group and URD group were mostly reduced by using original MDRD or CKD-EPI. Regarding the clinical outcomes, the number of hospitalized patients was most significantly reduced, as compared to critical illness, ICU need, death, or RRT. Fig 5 summarizes the clinical effects of renal variables on critical illness (defined as death or ICU need) in these sensitivity analyses. The effect of eGFR reduction tended to be more robust even with different formulas, than presence of AKI or URD. The similar tendencies were seen for the most of the clinical outcomes as shown in S3–S6 Tables, except for hospitalization in multivariate analyses, where eGFR reduction did not reach statistical significance.

**Table 6:** Comparison of patients with reduced ED eGFR based on different formulas.

Variable	eGFR formula		
	Japanese MDRD	Original MDRD	CKD-EPI
N (patients with eGFR < 60 in ED)^a^	441	230	223
Age, y, median (IQR)	77 (66.5, 84)	79 (67.75, 84)	80 (70, 84)
Sex, male (%)	243 (55.1)	129 (56.1)	131 (58.7)
**Renal function, median (IQR)**			
Baseline creatinine, mg/dL	1.04 (0.87, 1.33)	1.25 (0.97, 1.74)	1.26 (1.00, 1.80)
Baseline eGFR, mL/min/1.73m^2^	47.8 (38.6, 55.9)	51.7 (35.3, 63.9)	50.9 (33.0, 64.2)
ED creatinine, mg/dL	1.12 (0.94, 1.42)	1.41 (1.21, 1.89)	1.41 (1.21, 1.90)
ED eGFR, mL/min/1.73m^2^	45.7 (34.6, 53.3)	44.7 (31.0, 53.6)	43.7 (29.4, 52.9)
**Renal dysfunction category, n (%)**			
AKI only	22 (5.0)	23 (10.0)	23 (10.3)
AKI on CKD	32 (7.3)	23 (10.0)	24 (10.8)
CKD only	196 (44.4)	86 (37.4)	83 (37.2)
Subclinical kidney injury	27 (6.1)	26 (11.3)	22 (9.9)
URD	164 (37.2)	72 (31.3)	71 (31.8)
**Clinical outcome, n (%)**			
Death or ICU	86 (19.5)	62 (27.0)	62 (27.8)
Hospitalization	273 (61.9)	155 (67.4)	150 (67.3)
ICU need	73 (16.6)	52 (22.7)	51 (22.9)
Hospital death	25 (5.7)	19 (8.3)	20 (9.0)
RRT need	12 (2.7)	11 (4.8)	11 (4.9)

*eGFR*, estimated glomerular filtration rate; *ED*, emergency department; *ICU*, intensive care unit; *RRT*, renal replacement therapy; *AKI*, acute kidney injury; *URD*, undetermined renal dysfunction.

^a^ The number of patients whose eGFR (by each formula) < 60 mL/min/1.73m^2^ and were qualified for the full analysis.

**Fig 6:**
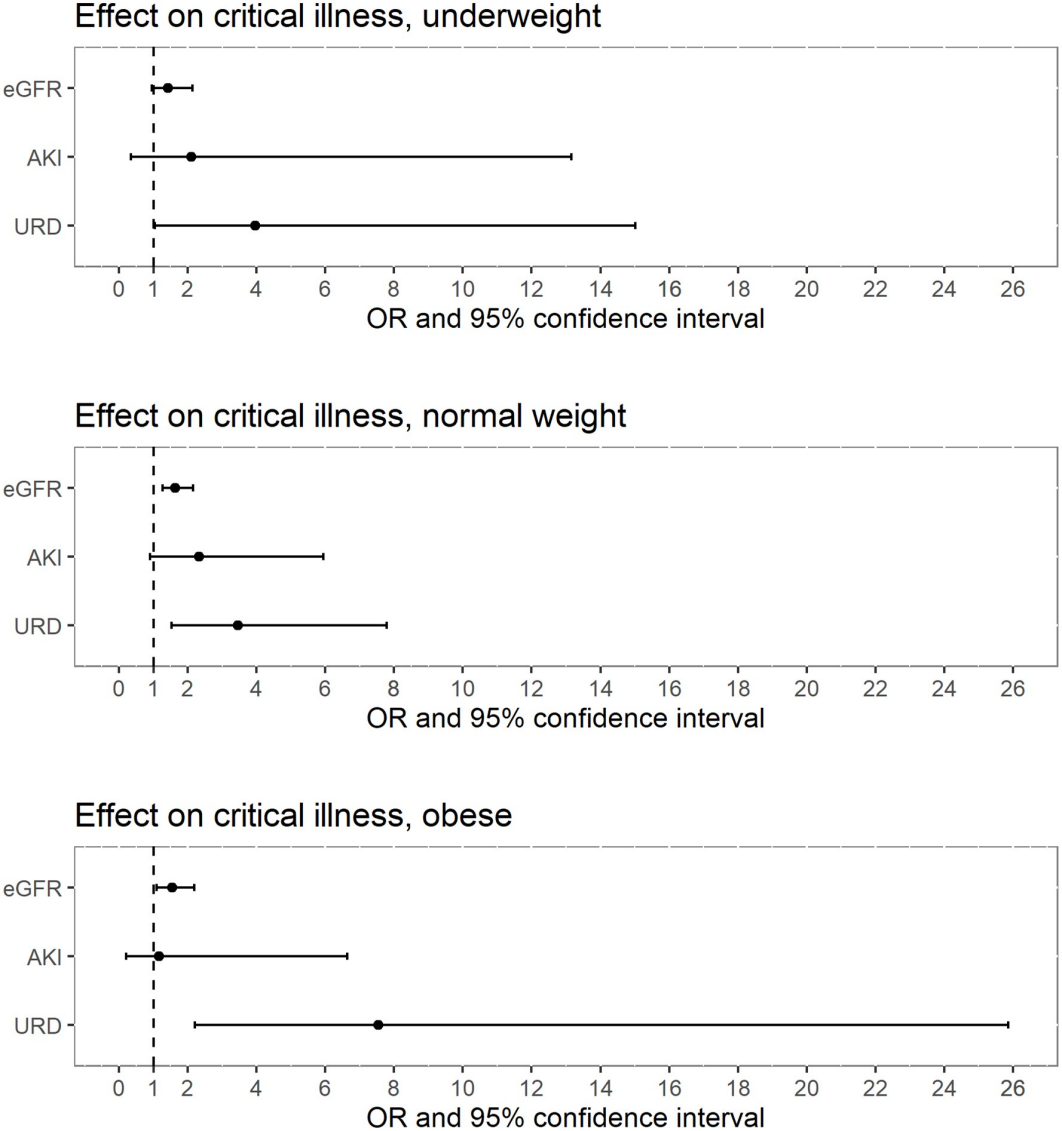
Effect sizes on critical illness by different BMI subgroups. Underweight, BMI < 18.5 kg/m^2^; normal weight, BMI of 18.5–25 kg/m^2^; obese, BMI ≧ 25 kg/m^2^. OR, odds ratio; eGFR, estimated glomerular filtration rate; AKI, acute kidney injury; URD, undetermined renal dysfunction; BMI, body mass index. For eGFR, OR for 10 mL/min/1.73m^2^ of eGFR decrease were shown.

The characteristics of BMI subgroups are summarized in Table 7. Interestingly, obese patients commonly required ICU care (29.2%) with only small proportion of death (1.4%). Death was most commonly seen in the underweight patients (12.3%, overall death rate was 5.8%). The results of the univariate logistic regression models by BMI subgroups are shown in Fig 6 and S7 Table. Reduction of eGFR, presence of AKI and URD seemed associated with increased odds of critical illness, although they did not necessarily reach statistical significance.

**Table 7:** Characteristics of BMI subgroups.

	Overall N = 329	Underweight (< 18.5 kg/m^2^) n = 65	Normal (18.5–25 kg/m^2^) n = 192	Obese (≧ 25 kg/m^2^) n = 72	p-value
**ED creatinine, mg/dL (IQR)**	1.15 (0.96, 1.47)	1.17 (0.96, 1.61)	1.12 (0.95, 1.41)	1.20 (1.01, 1.80)	0.1228
**ED eGFR, mL/min/1.73m**^**2**^ **(IQR)**	44.9 (33.4, 52.6)	43.8 (30.7, 53.2)	46.5 (35.1, 53.0)	43.2 (26.1, 51.7)	0.2408
**Death or ICU, no. (%)**	77 (23.4)	15 (23.1)	40 (20.8)	22 (30.6)	0.2508
**Hospitalization, no. (%)**	239 (72.6)	44 (67.7)	141 (73.4)	54 (75.0)	0.5874
**ICU need, no. (%)**	66 (20.0)	11 (16.9)	34 (17.7)	21 (29.2)	0.0914
**Hospital death, no. (%)**	19 (5.8)	8 (12.3)	10 (5.2)	1 (1.4)	**0.0264**
**RRT, no. (%)**	12 (3.6)	2 (3.1)	5 (2.6)	5 (6.9)	0.2686

Significant p-values are shown in bold.

*BMI*, body mass index; *IQR*, interquartile range; *ED*, emergency department; *ICU*, intensive care unit; *RRT*, renal replacement therapy; *eGFR*, estimated glomerular filtration rate.

Table 8 summarizes the area under the ROC curves to assess the performances of clinical factors measured at ED to predict clinical outcomes. Among them, the area under curves greater than 0.70 were observed in eGFR at ED visit for death and RRT need and GCS for death. The cutoffs of eGFR at ED visit for hospital death and RRT requirement were 28.9 mL/min/1.73m^2^ (sensitivity 0.44 and specificity 0.85) and 22.4 mL/min/1.73m^2^ (sensitivity 0.33 and specificity 0.91), respectively. When different formulas were used for eGFR calculation, these cutoffs changed as follows: eGFR by original MDRD at ED visit for prediction of hospital death and RRT requirement were 37.0 mL/min/1.73m^2^ (AUC 0.688 [0.553–0.798], sensitivity 0.74, specificity 0.69) and 27.7 mL/min/1.73m^2^ (AUC 0.740 [0.477–0.899], sensitivity 0.73, specificity 0.83), and eGFR by CKD-EPI at ED visit for prediction of hospital death and RRT requirement were 37.1 mL/min/1.73m^2^ (AUC 0.658 [0.515–0.778], sensitivity 0.70, specificity 0.67) and 28.7 mL/min/1.73m^2^ (AUC 0.723 [0.454–0.891], sensitivity 0.73, specificity 0.79), respectively.

**Table 8:** Area under receiver operating characteristic curve for clinical outcomes by continuous predictor variables.

AUC (95% CI)					
Predictor variable	Clinical outcome				
	Death or ICU	Hospitalization	ICU need	Hospital death	RRT need
Age	0.537 (0.467–0.606)	0.504 (0.449–0.561)	0.499 (0.426–0.572)	0.696 (0.570–0.798)	0.561 (0.377–0.730)
ED eGFR	0.696 (0.628–0.756)	0.611 (0.555–0.664)	0.679 (0.603–0.746)	* **0.728 (0.619–0.815)**	* **0.829 (0.666–0.921)**
SBP	0.600 (0.524–0.671)	0.559 (0.505–0.613)	0.563 (0.480–0.642)	0.699 (0.585–0.793)	0.646 (0.454–0.801)
BT	0.538 (0.461–0.612)	0.578 (0.524–0.631)	0.517 (0.439–0.595)	0.513 (0369–0.654)	0.438 (0.277–0.613)
GCS	0.666 (0.605–0.723)	0.559 (0.518–0.598)	0.632 (0.566–0.693)	* **0.738 (0.625–0.826)**	0.460 (0.322–0.604)
Platelet count	0.634 (0.562–0.701)	0.551 (0.497–0.605)	0.635 (0.560–0.703)	0.698 (0.564–0.806)	0.590 (0.433–0.731)
Total bilirubin	0.577 (0.502–0.648)	0.602 (0.549–0.653)	0.544 (0.465–0.620)	0.671 (0.533–0.785)	0.306 (0.154–0.517)

*AUC*, area under curve; *ICU*, intensive care unit; *RRT*, renal replacement therapy; *ED*, emergency department; eGFR, estimated glomerular filtration rate; *SBP*, systolic blood pressure; *BT*, body temperature; *GCS*, Glasgow coma scale.

* For those with AUC > 0.70, the best cutoffs using the Youden index are as follows: ED eGFR for death, 28.9 (sensitivity 0.44, specificity 0.85); ED eGFR for RRT need, 22.4 (sensitivity 0.33, specificity 0.91); and GCS for death, 14 (sensitivity 0.68, specificity 0.76).

## Discussion

The unique points of this study include the following: 1) all patients with eGFR < 60 mL/min/1.73m^2^ in ED were covered, including those who have no baseline renal function, and 2) both eGFR and acute changes of renal dysfunction were analyzed using AKI and CKD diagnosis criteria, and eGFR was concluded to be a more consistently reliable factor to predict poor outcomes compared with the evaluation of acute change of a renal function with the information of baseline serum creatinine values.

A previous study has proved that AKI in ED increased mortality rate in association with AKI severity [4]. However, it might be possible that what was considered as a clinical impact of AKI could partially be explained by the degree of eGFR reduction. Another study showed that reduced eGFR in a single measurement in ED was associated with higher mortality, although the acuteness of renal dysfunction was not covered [27]. To date, no study has directly compared the clinical importance of eGFR value and the acuteness of renal dysfunction in ED. In this study, both eGFR and acuteness of kidney function change were included in a multivariate logistic regression model to address possible confounding of eGFR on the acuteness of renal dysfunction or vice versa. The screening analysis in this study suggested the association between eGFR reduction and critical illness in both of younger and older population (Fig 2), which seems compatible with the previous study [27]. In addition, the obtained results from the full analysis have suggested that the clinical impact of AKI on ED outcomes partially might be derived from the degree of eGFR reduction and not necessarily from the acuteness of renal dysfunction (Table 5). Multivariate logistic regression in this study showed that the presence of AKI did not remain statistically significant in predicting the composite endpoint of death and ICU, hospitalization, or ICU need, whereas eGFR consistently showed a strong and statistically significant impact on those clinical outcomes. Although the multivariate analysis was not performed due to a relatively small number of positive cases, eGFR showed a strong association with hospital death and RRT need in univariate analysis. Since eGFR values are highly dependent on the type of formula, the robustness of these findings was further assessed in the sensitivity analyses (Table 6, S3–S6 Tables, Fig 5). The results were compatible with the original analyses. In addition, subgroup analysis by BMI categories was performed to evaluate the possible confounding effects of body habitus (Table 7, Fig 6, S7 Table), since BMI has been reported to affect both accuracy of GFR estimation [35] and the clinical outcomes [36, 37]. In terms of renal variables, the similar tendency to the original analyses was seen in these subgroups as well (Fig 6, S7 Table). Of note, the patients with lower BMI showed higher mortality rate in our analysis (Table 7), which is compatible with previous publications focusing on BMI [36, 37]. Recently, several studies have reported that eGFR is a useful marker in predicting clinical outcomes. For example, reduced eGFR was associated with higher 30-day mortality in ED [27]. Other studies showed that there were associations between lower eGFR and higher mortality in the patients with pneumonia [28] and acute heart failure [29]. In out-of-hospital cardiac arrest-related research, the patients with lower eGFR had a poorer survival rate [30]. Our findings support these previous reports. One possible limitation of our results is that the impact of the acuteness of renal dysfunction may have been underestimated because part of AKI had not been detected, and they were categorized into URD. However, the difficulty of identification of AKI from URD is an inevitable aspect of the clinical ED practice, as mentioned before, and a clinical decision has to be made with this limitation. According to our results, the process of predicting critical illness and mortality in ED can be simplified since physicians can rely solely on eGFR even when the acuteness of renal dysfunction cannot be immediately determined. Since a single measurement of serum creatinine can calculate eGFR, it can serve as the instantly available renal marker in ED, especially for patients they encountered for the first time.

In our study, all patients with a renal dysfunction based on a low eGFR in ED were enrolled. Those without the information of baseline serum creatinine were also included since one of our goals was to describe the epidemiology of renal dysfunction in the real-world setting of ED. Physicians may not have their patients’ baseline renal function in a common ED situation and need to see patients with elevated serum creatinine or reduced eGFR of unknown acuteness [26]. The characteristics of renal dysfunction of the different categories, AKI only, CKD only, AKI on CKD, subclinical kidney injury, and URD, have been elaborated in this study. As expected from the previous publications [2–9, 21–23], the AKI patients in the AKI only and the AKI on CKD groups had relatively higher mortality rates. However, they represented only 12.2% of overall patients in our study. Conversely, more than 30% of the patients with renal impairment had no baseline data and were categorized into the URD category (Table 3). The results obtained in this study have suggested that URD might be composed of a highly heterogeneous population with mixed severity, and their response to treatment was generally good. For example, URD patients more frequently required ICU treatment than the other categories (Table 3), which suggests that URD may represent a relatively sicker population. However, simultaneously, the hospitalization rate of URD was not higher than overall patients, and mortality was actually low (3.0%) (Table 3). With the high rate of URD, these patients might have had a considerable influence on ED statistics and the generalizability of previous clinical studies focusing on AKI.

For the actual clinical use of eGFR in ED, the area under ROC curves of continuous independent variables has been calculated to predict different clinical outcomes (Table 8). Among all, eGFR in ED had a statistically and clinically significant level of accuracy (area under curve > 0.70) to predict death and RRT need. The calculated best cutoffs of eGFR were 28.9 mL/min/1.73m^2^ and 22.4 mL/min/1.73m^2^ to predict death and RRT need, respectively. Interestingly, the calculated best cutoff for the prediction of death was 28.9 mL/min/1.73m^2^, which is close to 30 mL/min/1.73m^2^, the cutoff between CKD stages G3 and G4 [25]. The cutoffs to predict death by different formulas were 37.0 mL/min/1.73m^2^ for original MDRD and 37.1 mL/min/1.73m^2^ for CKD-EPI, respectively. Although these are somewhat higher than the cutoff by Japanese MDRD, they are still close to the borderline of CKD stages G3 and G4. A remarkable increase of mortality in a comparison between CKD stages G3 and G4 has been reported [38, 39].

Nonrenal variables also showed associations with the clinical outcomes to various degrees (Tables 4 and 5). For example, high body temperature and low platelet count were both associated with the clinical outcomes in our study. They are known to be associated with adverse outcomes in ED and ICU [40–43], and various causes have been proposed as possible mechanisms in these responses, including sepsis, drug-induced reaction, and environmental illness such as heat stroke. These conditions can influence multiple organ systems, including kidneys. Although we did not focus on the detailed causes of each patient’s renal dysfunction in this study, the etiology specific interaction of organ failure and clinical outcomes will be a quite interesting, possible future step.

This study has several limitations. This is a single-center retrospective study with a limited number of patients, and the generalizability of the findings may be affected. Many patients were not qualified for the full analysis, either due to lack of eGFR measurement (1317 patients) or ED eGFR ≧ the cutoff value, 60 mL/min/1.73m^2^ (1027 patients). Although the majority of the patients with primary outcome (critical illness defined as death or ICU need) tended to have eGFR < 60 mL/min/1.73m^2^ and were included in the full analysis (Table 1), the results of the study have to be carefully applied to clinical practice. The cohort mainly analyzed in this study may represent relatively older and more severely ill patients. Additionally, the numbers of positive outcomes were not always sufficient for the statistical analysis. For example, in-hospital death was only 25 in this study, which was too small for multivariate analysis. The number of RRT was also insufficient, and multivariate analysis was not performed either. As compared to the conclusions on critical illness, those on death or RRT may be less reliable. Finally, there is a difficulty in evaluating patients’ renal dysfunction with a single measurement of eGFR. In patients with AKI, various formulas for GFR estimation may not accurately reflect the true GFR [44, 45]. However, even with this difficulty, previous publications have suggested that an eGFR in ED may serve as a predictor of clinical outcomes [27–30] and our results support these findings. Lastly, the definition of “baseline” renal function, the last value between 7 days and 1 year before the ED visit, might not represent the real baseline for all the patients.

## Conclusions

Among ED patients with impaired renal function, eGFR was illustrated to be an independent predictor of critical illness defined as death or ICU need, hospitalization, and ICU need, even after adjustment with AKI or URD. Estimated GFR also had a strong association with hospital death and RRT need. URD was a very common finding among those with renal dysfunction in ED, which may be a highly heterogeneous condition influencing ED statistics. A single measurement of eGFR might be sufficient information in the ED, regardless of AKI complication.

## Supporting information

S1 Table Data of the screened patients. (XLSX)

S2 Table Data of the patients enrolled in the full analysis. (XLSX)

S3 Table Sensitivity analysis: Univariate logistic regression model for factors associated with clinical outcomes (eGFR calculated with original MDRD). (DOCX)

S4 Table Sensitivity analysis: Univariate logistic regression model for factors associated with clinical outcomes (eGFR calculated with CKD-EPI). (DOCX)

S5 Table Sensitivity analysis: Multivariate logistic regression model for factors associated with clinical outcomes (eGFR calculated with original MDRD). (DOCX)

S6 Table Sensitivity analysis: Multivariate logistic regression model for factors associated with clinical outcomes (eGFR calculated with CKD-EPI). (DOCX)

S7 Table Subgroup analysis: Univariate logistic regression models for death or ICU by BMI subgroups. (DOCX)
