# Supplementary material for: Estimated glomerular filtration rate may be an independent predictor for clinical outcomes regardless of acute kidney injury complication in the emergency department
Source: PLoS One. 2021 Oct 14;16(10):e0258665. doi: 10.1371/journal.pone.0258665 (PMC8516290; doi:10.1371/journal.pone.0258665)
Supplement: S6 Table — (DOCX) [file pone.0258665.s006.docx]

**S6 Table. Sensitivity analysis: multivariate logistic regression model for factors associated with clinical outcomes (eGFR calculated with CKD-EPI).**

| Outcome,  positive cases (%) | Patient factor | Reference | p-value | OR (95% CI) |
| --- | --- | --- | --- | --- |
| **Death or ICU**  **62 (27.8)** | AKI-positive | AKI-negative | 0.9616 | 0.98 (0.40–2.41) |
|  | URD | AKI-negative | 0.4513 | 1.35 (0.62–2.95) |
|  | eGFR | N/A^a^ | **0.0009** | 1.49 (1.17–1.88) |
|  | Platelet count < 150 × 10^3^/μL | Platelet count ≥150 × 10^3^/μL | **0.0065** | 2.81 (1.34–5.92) |
|  | Total bilirubin ≥1.2 mg/dL | Total bilirubin < 1.2 mg/dL | 0.2212 | 1.72 (0.72–4.09) |
|  | GCS < 15 | GCS = 15 | **<0.0001** | 4.75 (2.32–9.75) |
|  | Age ≥75 | Age < 75 | 0.2700 | 0.67 (0.33–1.37) |
| **Hospitalization**  **150 (67.3)** | AKI-positive | AKI-negative | 0.1048 | 2.01 (0.86–4.70) |
|  | URD | AKI-negative | 0.1092 | 1.74 (0.88–3.44) |
|  | eGFR | N/A^a^ | 0.0887 | 1.21 (0.97–1.51) |
|  | Platelet count < 150 × 10^3^/μL | Platelet count ≥150 × 10^3^/μL | 0.4099 | 1.35 (0.66–2.76) |
|  | Total bilirubin ≥1.2 mg/dL | Total bilirubin < 1.2 mg/dL | 0.0565 | 2.46 (0.98–6.21) |
|  | GCS < 15 | GCS = 15 | 0.0839 | 1.85 (0.92–3.70) |
|  | Age ≥75 | Age < 75 | 0.4037 | 0.76 (0.41–1.44) |
| **ICU need**  **51 (22.9)** | AKI-positive | AKI-negative | 0.8208 | 1.11 (0.44–2.84) |
|  | URD | AKI-negative | 0.0619 | 2.14 (0.96–4.78) |
|  | eGFR | N/A^a^ | **0.0010** | 1.49 (1.17–1.91) |
|  | Platelet count < 150 × 10^3^/μL | Platelet count ≥150 × 10^3^/μL | **0.0192** | 2.51 (1.16–5.44) |
|  | Total bilirubin ≥1.2 mg/dL | Total bilirubin < 1.2 mg/dL | 0.8897 | 0.94 (0.37–2.37) |
|  | GCS < 15 | GCS = 15 | **0.0043** | 2.90 (1.40–6.01) |
|  | Age ≥75 | Age < 75 | 0.3036 | 0.68 (0.32–1.42) |

Significant p-values are shown in bold.

*OR*, odds ratio; *CI*, confidence interval; *ICU*, intensive care unit; *RRT*, renal replacement therapy; *AKI*, acute kidney injury; *URD*, undetermined renal dysfunction; *eGFR*, estimated glomerular filtration rate; *GCS*, Glasgow Coma Scale

AKI-positive means those who were proven to have AKI, whereas AKI-negative means those who were proven NOT to have AKI based on the KDIGO guidelines. URD are the patients who could not be diagnosed with or ruled out AKI due to lack of an information on their baseline renal function.

^a^ For eGFR, OR for 10 mL/min/1.73m^2^ of eGFR decrease were shown.
